# Current generation time-of-flight ^18^F-FDG PET/CT provides higher SUVs for normal adrenal glands, while maintaining an accurate characterization of benign and malignant glands

**DOI:** 10.1007/s12149-015-1041-z

**Published:** 2015-12-07

**Authors:** Daniëlle Koopman, Jorn A. van Dalen, Jos A. Stigt, Cornelis H. Slump, Siert Knollema, Pieter L. Jager

**Affiliations:** Department of Nuclear Medicine, Isala, Dokter van Heesweg 2, 8025 AB Zwolle, The Netherlands; MIRA Institute for Biomedical Technology and Technical Medicine, University of Twente, Enschede, The Netherlands; Department of Medical Physics, Isala, Zwolle, The Netherlands; Department of Pulmonology, Isala, Zwolle, The Netherlands

**Keywords:** ^18^F-FDG, PET/CT, Adrenal glands, Lung cancer

## Abstract

**Objective:**

Modern PET/CT scanners have significantly improved detectors and fast time-of-flight (TOF) performance and this may improve clinical performance. The aim of this study was to analyze the impact of a current generation TOF PET/CT scanner on standardized uptake values (SUV), lesion-background contrast and characterization of the adrenal glands in patients with suspected lung cancer, in comparison with literature data and commonly used SUV cut-off levels.

**Methods:**

We included 149 adrenal glands from 88 patients with suspected lung cancer, who underwent ^18^F-FDG PET/CT. We measured the SUV_max_ in the adrenal gland and compared this with liver SUV_mean_ to calculate the adrenal-to-liver ratio (AL ratio). Results were compared with literature derived with older scanners, with SUV_max_ values of 1.0 and 1.8 for normal glands [1, 2]. Final diagnosis was based on histological proof or follow-up imaging. We proposed cut-off values for optimal separation of benign from malignant glands.

**Results:**

In 127 benign and 22 malignant adrenal glands, SUV_max_ values were 2.3 ± 0.7 (mean ± SD) and 7.8 ± 3.2 respectively (*p* < 0.01). Corresponding AL ratios were 1.0 ± 0.3 and 3.5 ± 1.4 respectively (*p* < 0.01). With a SUV_max_ cut-off value of 3.7, 96 % sensitivity and 96 % specificity was reached. An AL ratio cut-off value of 1.8 resulted in 91 % sensitivity and 97 % specificity. The ability of both SUV_max_ and AL ratio to separate benign from malignant glands was similar (AUC 0.989 vs. 0.993, *p* = 0.22).

**Conclusions:**

Compared with literature based on the previous generation of PET scanners, current generation TOF ^18^F-FDG PET/CT imaging provides higher SUVs for benign adrenal glands, while it maintains a highly accurate distinction between benign and malignant glands. Clinical implementation of current generation TOF PET/CT requires not only the use of higher cut-off levels but also visual adaptation by PET readers.

## Introduction

Metastatic spread in non-small cell lung cancer (NSCLC) typically involves the brain, bone, liver, contralateral lung and adrenal glands [3]. An accurate evaluation of the adrenal glands is important, especially when the glands are enlarged on computed tomography (CT) [4, 5].

The adrenal glands can be characterized with several imaging techniques, like CT, magnetic resonance imaging (MRI), positron emission tomography (PET) using the tracer fluorine-18 fluordeoxyglucose (^18^F-FDG) and combined ^18^F-FDG PET/CT. All these techniques are capable of distinguishing benign from malignant adrenal masses, although with different degrees of accuracy [6]. Especially ^18^F-FDG PET/CT performs well and is often used for this purpose as it is part of standard clinical practice in NSCLC.

The combination of the small size of the normal adrenal gland with the low ^18^F-FDG uptake make the glands usually invisible on older PET scanners because of relatively low resolution. In 2004, a paper about the appearance of the normal adrenal gland on ^18^F-FDG PET was published, which reported an average maximum standardized uptake value (SUV_max_) around 1.0 [1]. More recently, Kim et al. reported an average SUV_max_ value around 1.8 for normal glands [2]. The introduction of combined PET/CT made the evaluation of adrenal glands with ^18^F-FDG PET easier because the location of tracer uptake could now be more accurately assigned to the adrenal gland as seen on CT [1]. The visual aid provided by CT therefore led to a larger proportion of gland visualization, although ^18^F-FDG uptake was still similar to the PET/CT era. Furthermore, it was demonstrated that the use of combined PET/CT improved the distinction between benign and malignant adrenal masses [7, 8].

In the past decade, however, PET technology itself has been upgraded with new, faster scintillators like Lutetium-Orthosilicate (LSO) and Lutetium-Yttrium-Orthosilicate (LYSO). As a consequence, time-of-flight (TOF) PET became the new standard technology for PET manufacturers. Generally, incorporation of the TOF technique leads to a more accurate determination of the origin of the annihilation event [9], resulting in improved measurements of ^18^F-FDG uptake.

Even more recently, TOF performance is steadily improving in newer scanners. A recently introduced TOF PET/CT scanner (Ingenuity TF, Philips Healthcare) has a time-of-flight performance with a timing resolution of around 500 picoseconds [10], which translates to a location uncertainty of 7.5 cm on lines-of-response. The improved temporal resolution induces a higher signal-to-noise ratio, higher spatial resolution and improved PET image quality [11].

Therefore, the current generation of PET scanners may lead to a better detection of the adrenal glands as well as other small lesions. Therefore, the aim of this investigation was to analyze the impact of a current generation TOF PET/CT scanner on SUVs, lesion-to-background levels and characterization of adrenal glands in patients with clinically suspected lung cancer.

## Materials and methods

### Patients

We included 88 patients, referred for a whole-body ^18^F-FDG PET/CT scan, to evaluate clinically suspected lung cancer. Only patients harboring at least one adrenal gland with a final diagnosis, based on histological proof or follow-up imaging, were selected for this study. For 27 patients who underwent a single adrenal gland biopsy, no follow-up imaging was available resulting in an unknown status for the contralateral gland that was not invasively evaluated. Consequently, in these patients, only the adrenal gland with histological proof was included.

We received a waiver from the Medical Ethical Committee of our institution to perform this partly retrospective and prospective study, as it only deals with additional evaluation of a clinical indicated scan. However, all patients agreed to the use of their data by signing an informed consent form.

### PET/CT data acquisition

Patients fasted for at least 6 h prior to scanning. Before intravenous injection of ^18^F-FDG, blood glucose levels were measured to ensure a value below 15 mmol/L. A dedicated dose protocol depending quadratically on patients’ body weight, as recently proposed in the literature [12], was routinely used. It is described by the formula *A* × *t* = 3.8 × *w*^2^, where *A* is the ^18^F-FDG dose to administer (in MBq), *t* the time per bed position (in seconds) and *w* is the patients’ body weight (in kilogram).

All PET/CT scans were acquired with a current generation PET/CT scanner (Ingenuity TF, Philips Healthcare, Cleveland, OH, USA). This fully three-dimensional TOF scanner is combined with a 128-slice CT scanner. The PET system contains 28,336 LYSO crystals (size 4 × 4 × 22 mm) divided across 44 detector rings. Regarding TOF performances, the timing resolution of the PET scanner is 495 picoseconds with a TOF localization-accuracy of 7.4 cm. The PET scan was performed using a whole-body PET/CT acquisition protocol with 50 % bed-overlap. Acquisition times for the patient studies were 1 and 2 min per bed position for patients with body weight ≤80 and >80 kg, respectively. The average administered ^18^F-FDG activity was 330 MBq (range 154–557 MBq).

Prior to PET imaging, a CT scan was acquired for attenuation correction. The CT scan parameters were: tube voltage 120 kV, average tube current 61 mA (range 36–140 mA), slice collimation 64 × 0.625 mm, pitch 0.83 and rotation time 0.5 s. The average CT dose-index was 4.1 mGy (range 2.5–9.3) with an average dose-length product of 438 mGy cm (range 250–930).

### PET/CT data reconstruction

PET data were reconstructed using the default reconstruction algorithm “Blob-OS-TF”, a 3D ordered subset iterative TOF reconstruction technique [13, 14]. For all reconstructions, 3 iterations and 43 subsets were applied. PET images were reconstructed in 144 × 144 matrices with voxel size 4 × 4 × 4 mm^3^ and relaxation parameter 1.0. The blob had a 2.5 mm radius with a blob shape parameter of 8.4 mm. Point-spread function modeling was not applied.

Raw CT data were reconstructed using an iterative reconstruction algorithm (iDose, Philips Healthcare, Cleveland, OH, USA) with iDose level 4 and a slice thickness of 3 mm. The acquisition- and reconstruction protocol were both compatible with the guidelines of the European Association of Nuclear Medicine (EANM) [15].

### PET/CT data analysis

Integrated PET/CT data were reviewed on a dedicated workstation (IntelliSpace Portal 6, Philips Healthcare, Cleveland, OH, USA). The attenuation CT scan was used to identify the adrenal glands and to characterize the gland as normal sized or enlarged. The adrenal gland was regarded as enlarged when an adrenal mass of at least 1 cm was detected on an axial CT slice.

When histological evidence was not available, adrenal glands were considered benign when they were stable in size and had identical ^18^F-FDG uptake on follow-up imaging. Adrenal glands were considered malignant when there was progression in size or ^18^F-FDG uptake had increased as evaluated by experienced readers. Furthermore, in patients who received systemic treatment during the follow-up period, glands were considered malignant when they showed a decrease in size or ^18^F-FDG uptake on follow-up imaging.

Quantitative PET measurements were performed by an experienced PET reader, blinded to histological and follow-up findings, who measured the uptake on the axial slice through the adrenal with the highest visual uptake. Around the adrenal, an elliptical 2D region-of-interest (ROI) was drawn that included at least two-thirds of the gland. After accurate placement of the adrenal ROI was confirmed on axial PET/CT images, the SUV_max_ within the ROI was determined.

Furthermore, we performed background measurements in a homogenous region in the liver. On the axial PET image containing the largest liver area, we drew a circular 2D ROI of 2000 mm^2^ (5 cm diameter) in the right liver lobe and measured the mean standardized uptake value (SUV_mean_) within this ROI. We made sure that this liver section was homogeneous on ^18^F-FDG PET and free of any tumor or benign abnormality. For each adrenal gland, the adrenal-liver ratio (AL ratio) was derived by dividing adrenal gland SUV_max_ by liver SUV_mean_.

### Data analysis

We calculated average SUV_max_ and AL ratio values for benign and malignant adrenal glands, including a sub-analysis for normal-sized and enlarged glands. We determined the sensitivity and specificity of SUV_max_ and AL ratio for adrenal gland characterization at various cut-off levels, using the final diagnosis as a reference standard. For both parameters, the area under the curve (AUC) with the 95 % confidence-interval (CI) was provided.

### Statistical analysis

Results were presented as mean ± standard deviation (SD). Furthermore, ranges (minimum–maximum) in uptake values were included. We applied the Independent samples Mann–Whitney *U* test to compare the SUV_max_ and AL ratio between benign and malignant glands and normal-sized and enlarged glands, respectively. To evaluate differences in characterization performance between SUV_max_ and AL ratio, we compared the AUCs for both parameters using a Chi-square test. A *p* value less than 0.05 was considered to indicate statistical significance.

## Results

### Patient characteristics

Clinical data from 88 patients and 149 evaluated adrenal glands are presented in Table 1. All adrenal glands were identified on PET/CT images. There were 22 malignant and 127 benign glands. 91 % (20/22) of the malignant glands were enlarged. Conversely, 40 % (20/50) of the enlarged glands were malignant. For 76 % of the adrenal glands (113/149) the final diagnosis was based on follow-up imaging. The average follow-up period was 6.1 months, which is fairly long in relation to the general rate of progression of lung cancer.

**Table 1:** Summary of patient characteristics

Patients characteristics (*n* = 88)	
Sex	
Male	56
Female	32
Age (years)	66 ± 11
Bodyweight (kg)	78 ± 15
Glucose (mmol/L)	6.1 ± 1.4
Follow-up period (months)	6.1 ± 2.4
Adrenal glands (*n* = 149)	
Final diagnosis	
Benign	127
Malignant	22
Final diagnosis from	
Histological proof	36
Follow-up imaging	113
Gland characterization	
Normal-sized	99
Enlarged	50
Enlarged glands (*n* = 50)	
Benign	30
Malignant	20

### Average uptake values for benign and malignant glands

**Fig. 1:**
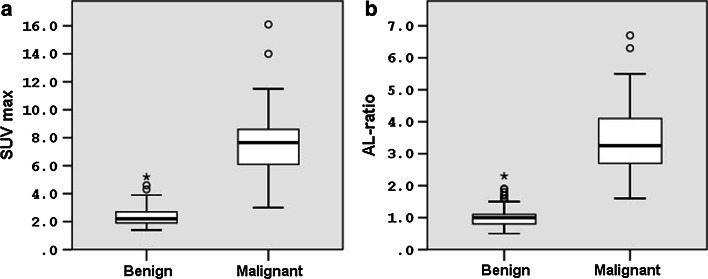
Box plots of the adrenal gland SUV_max_ (**a**) and AL ratios (**b**) for benign and malignant adrenal glands. *Significant difference (*p* < 0.001) in SUV_max_ and AL ratio between benign and malignant glands. *Circle o* values that are between 1.5 and 3.0 *box* length from the percentile borders. *Asterisk* values that fall more than 3.0* box* length outside the* box* borders

Table 2 contains average SUV_max_ and AL ratios for benign (*n* = 127) and malignant (*n* = 22) adrenal glands. For both parameters, we found significantly higher uptake values for malignant glands as compared to benign glands (*p* < 0.001), but with a wide range in uptake values for both groups as visualised in Fig. 1.

**Table 2:** SUV_max_ and AL ratios for benign and malignant adrenal glands

	Benign glands (*n* = 127)	Malignant glands (*n* = 22)	*p* value
SUV_max_			
Mean ± SD	2.3 ± 0.7	7.8 ± 3.2	*p* < 0.001
Range	1.4–5.2	3.0–16.1	
AL ratio			
Mean ± SD	1.0 ± 0.3	3.5 ± 1.4	*p* < 0.001
Range	0.5–2.3	1.6–6.7

### Normal-sized and enlarged adrenal glands

In Table 3, average SUV_max_ and AL ratios for normal-sized (*n* = 99) and enlarged (*n* = 50) glands are presented. In enlarged glands, we found significantly higher uptake values when they were malignant (*p* < 0.001). In normal-sized malignant glands, we also observed higher uptake values as compared to normal-sized benign glands, although the number of normal-sized malignant glands was limited (*n* = 2).

**Table 3:** SUV_max_ and AL ratios for normal-sized and enlarged, benign and malignant glands

	Normal-sized glands		*p* value	Enlarged glands		*p* value
	Benign (*n* = 97)	Malignant (*n* = 2)		Benign (*n* = 30)	Malignant (*n* = 20)	
SUV_max_						
Mean ± SD	2.3 ± 0.6	5.5 ± 0.8	*p* < 0.001	2.6 ± 0.7	8.0 ± 3.3	*p* < 0.001
Range	1.4–4.6	4.9–6.1		1.4–5.2	3.0–16.1	
AL ratio						
Mean ± SD	1.0 ± 0.3	2.8 ± 0.6	*p* < 0.001	1.3 ± 0.3	3.6 ± 1.4	*p* < 0.001
Range	0.5–1.9	2.4–3.3		0.8–2.3	1.6–6.7	

**Fig. 2:**
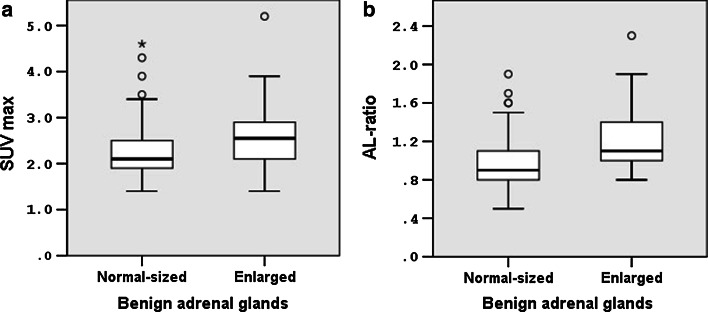
Box plots of the adrenal gland SUV_max_ (**a**) and AL ratios (**b**) for normal-sized and enlarged benign adrenal glands. *Significant difference in SUV_max_ (*p* = 0.01) and AL ratio (*p* < 0.001) between normal-sized and enlarged benign glands. *Circle o* values that are between 1.5 and 3.0* box* length from the percentile borders. *Asterisk* values that fall more than 3.0* box* length outside the* box* borders

Furthermore, for enlarged benign glands there were slightly higher uptake values as compared to normal-sized benign glands (*p* = 0.01 for SUV_max_ and *p* < 0.001 for AL ratio, respectively). For both groups of benign glands, there was a wide range in uptake values as visualised in Fig. 2.

### Adrenal gland visualization with ^18^F-FDG PET/CT

**Fig. 3:**
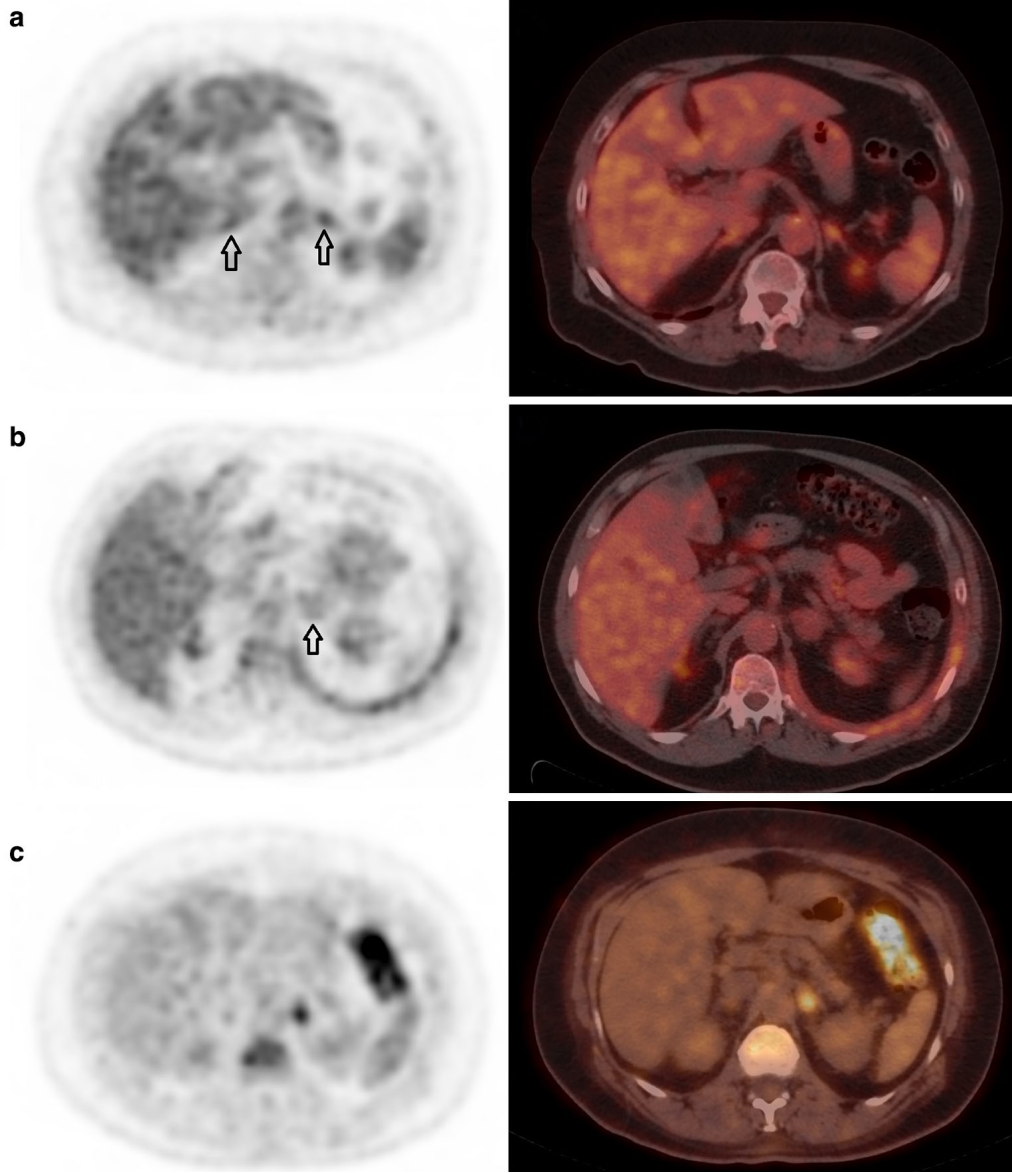
Single ^18^F-FDG PET (left) and fused ^18^F-FDG PET/CT (*right*) images of three patients. **a** Two normal-sized adrenal glands (*arrows*) are visualised. Uptake values for the left adrenal gland were 3.4 (SUV_max_) and 1.2 (AL ratio). For the right adrenal gland, the SUV_max_ and AL ratio were 3.2 and 1.1 respectively. A 6-months follow-up ^18^F-FDG PET/CT scan did not reveal any changes in adrenal gland size, shape and ^18^F-FDG uptake for both adrenal glands. **b** Patient with an enlarged left adrenal gland (*arrow*), axial diameter 32 mm. Average uptake values were 2.6 (SUV_max_) and 1.0 (AL ratio). A biopsy performed during endoscopic ultrasound was negative for tumor cells. Furthermore, 3-month follow-up ^18^F-FDG PET/CT imaging did not reveal metastatic disease in this enlarged adrenal gland. **c** Patient with a metastatic lesion in the left adrenal gland. On ^18^F-FDG PET images, we measured a SUV_max_ of 4.9 and an AL ratio of 3.3 for this adrenal gland. A biopsy performed during endoscopic ultrasound-guided fine-needle aspiration (EUS-FNA) confirmed the presence of an adrenal metastasis, originating from a lung tumor

Figure 3 contains ^18^F-FDG PET/CT images of three patients with benign normal-sized, benign enlarged and malignant adrenal glands. These examples present the typical visualization of adrenal glands as visualised on ^18^F-FDG TOF PET/CT imaging.

### Adrenal gland characterization

Both the SUV_max_ and the AL ratio provided an accurate separation of benign from malignant glands. For the SUV_max_, a cut-off value of 3.7 resulted in both 96 % sensitivity and specificity. Using a lower SUV_max_ of 3.0, 100 % sensitivity was reached. However, this resulted in only 84 % specificity. Furthermore, a higher SUV_max_ of 5.7 resulted in 100 % specificity with 77 % sensitivity.

For the AL ratio, a cut-off value of 1.8 resulted in 91 % sensitivity and 97 % specificity. Using a lower AL ratio of 1.6, 100 % sensitivity was reached with 92 % specificity. Furthermore at AL ratio 2.4, 100 % specificity was reached with 86 % sensitivity.

The Chi-square test showed that the ability of both SUV_max_ and AL ratio to separate benign from malignant glands was similar (*p* = 0.22) with AUC values 0.989 (CI 0.974–1.000) and 0.993 (CI 0.983–1.000), respectively.

## Discussion

The present study shows that the use of current generation TOF PET/CT imaging yields higher SUVs in benign adrenal glands, as compared to values described in the literature based on the previous generation of PET scanners. Consequently, the visualization of normal glands on current generation TOF PET/CT has improved. Although theoretically the improved visualization of benign glands may lead to confusion, it appeared that the ability of ^18^F-FDG PET to distinguish between benign and malignant nature of the adrenal lesions remains very high, as SUVs in malignant glands is also significantly higher than before. Using appropriate cut-off values, 96 % sensitivity and 96 % specificity can be reached, which leads to an accurate detection of metastatic adrenal gland lesions with modern PET/CT.

This study can be regarded as an update of published results in adrenal glands based on older PET/CT technology. Results from the present study differ in many aspects from previous investigations described in literature. For example, we found statistically different SUVs between normal-sized and enlarged benign glands (*p* = 0.01) which has been described before; however, in our study the mean difference in SUV between these groups was relatively small (SUV 2.3 vs. SUV 2.6). For enlarged adrenal glands (also mentioned adrenal lesions, nodules or masses), it is known that they may show an increased ^18^F-FDG uptake when benign [16, 17] and our results are comparable with values reported in the literature. However, for normal-sized glands, an average SUV_max_ of 2.3 as found in our study is considerably higher than values of 1.0 and 1.8 as reported in the literature [1, 2]. In other words, with current generation TOF PET/CT there is an increase in measured SUV, especially for normal-sized benign glands. Their SUVs get closer to the commonly reported uptake values for enlarged benign glands.

Furthermore, our study revealed a broad range (1.4–5.2) in SUV_max_ for benign adrenal glands. The presence of a wide range in uptake values has been mentioned before [1, 2], but with much smaller ranges (0.95–2.46 and 1.0–3.3, respectively). Higher SUVs in normal-sized benign adrenal glands, as found in the present study, are likely caused by the improved resolution of current PET cameras with fast TOF providing a better image quality for more accurate SUV measurements [18]. Also the reduced partial volume effect contributes to the more accurate evaluation of small organs such as the adrenals.

In simplified terms, using older generation of PET scanners a clearly visualized adrenal could often times safely and correctly be reported as malignant. With the modern scanners, however, this is no longer the case. PET readers should be aware of higher SUVs as well as wider ranges in benign adrenal glands when evaluating scans acquired with current generation TOF PET/CT. Readers should also update their cut-off levels if they decide to use SUV measurements to assist the visual diagnosis. It is expected that this new particular knowledge improves the diagnostic confidence of PET readers and prevents unnecessary adrenal gland biopsies when implementing current generation TOF PET/CT.

The main advantage of ^18^F-FDG PET/CT in adrenal gland evaluation is the ability to detect and exclude metastatic disease. Therefore, our finding that normal adrenal glands show higher ^18^F-FDG uptake values on TOF PET/CT may make interpretation more difficult, as it can be hypothesized that the presence of higher SUVs in normal adrenal glands makes the distinction between benign glands and (small) adrenal metastases more difficult. However, in our study uptake in malignant glands was also considerably higher and overall, we showed that ^18^F-FDG PET/CT still has a great ability to detect metastatic disease in adrenal glands, with 96 % sensitivity and 96 % specificity at a SUV_max_ cut-off level of 3.7. Many studies described lower SUV_max_ cut-off values ranging between 2.5 and 3.4 [2, 7, 19–22]. Furthermore, one study reported an optimal SUV_max_ of 3.9 with a relative poor performance (sensitivity 96 %, specificity 82 %) [23] while another study recently found sensitivity and specificity values of 90 % using SUV_max_ cut-off 5.2 [18].

Apart from the use of SUV_max_ in image interpretation, adrenal gland uptake is always visually or quantitatively compared with tracer intensity in the liver. In the present study, SUV_max_ and AL ratio performances for adrenal gland characterization were comparable. Nonetheless, some studies suggested that the AL ratio is the most accurate parameter for gland characterization [18, 23, 24]. In the past 15 years, many studies used a visual or quantitative AL ratio equal to or larger than 1.0 as cut-off value to separate benign from malignant adrenal glands [5, 8, 24, 25]. In contrast, our study indicates that an AL ratio of at least 1.6 should be used for accurate distinction while recently, two other studies reported AL ratios of 1.4 and 1.5 with sensitivity and specificity values above 90 % [18, 23]. Hence, this demonstrates that clinical implementation of current generation TOF PET/CT requires higher AL ratio cut-offs than used to the past, which demands a change in both quantitative and visual adrenal gland assessment by PET readers.

In this study we demonstrated that the current generation TOF PET/CT scanner is highly accurate in adrenal gland characterization. However, we also found a wide range in benign gland SUVs with some overlap in malignant gland SUVs. Therefore, when a gland with a SUV ranging between 3 and 6 is suspected to be the only metastatic location in a patient, the status is still often verified by biopsy (for left-sided glands) or short term follow-up imaging (for right-sided glands), despite the very high detection performance of PET.

The present study has some limitations. With an average follow-up of 6 months, the follow-up period in this study may be short, potentially biasing the results. However, on average NSCLC rapidly progresses even after chemotherapy. It seems unlikely that a normal sized metastatic gland remains unchained in this period, although we cannot completely exclude this possible small error. Furthermore, the diagnostic performance might be further improved if we would take additional information such as adrenal gland Hounsfield measurements and contrast washout into account [5, 7, 21]. Moreover, we focused on quantitative assessment of the adrenal glands, while visual adrenal-to-background evaluation on ^18^F-FDG PET remains important and sensitive in clinical practice [19, 26]. Finally, adrenal gland uptake may differ depending on the populations studied [2].

## Conclusion

Current generation TOF ^18^F-FDG PET/CT imaging results in higher SUVs and adrenal-to-liver ratios in benign adrenal glands, as compared to literature based on older scanners. This leads to an improved visualization of both benign and malignant glands and requires adaptation of PET readers when evaluating current generation TOF PET/CT images. However, this study indicates that the quantitative differentiation between benign and malignant glands in patients with suspected lung cancer remains highly accurate, when appropriate cut-off levels are used.
